# A randomised trial of Acceptance and Commitment Therapy for Anorexia Nervosa after daycare treatment, including five-year follow-up

**DOI:** 10.1186/s12888-016-0975-6

**Published:** 2016-07-29

**Authors:** Thomas Parling, Martin Cernvall, Mia Ramklint, Sven Holmgren, Ata Ghaderi

**Affiliations:** 1 Department of Psychology, Uppsala University, P.O. Box 1225, SE-75142 Uppsala, Sweden; 2 Department of Public Health and Caring Sciences, Uppsala University, Uppsala, Sweden; 3 Department of Neuroscience, Psychiatry, Uppsala University, Uppsala, Sweden; 4 Uppsala University Hospital, Uppsala, Sweden; 5 Department of Clinical Neuroscience, Karolinska Institutet, Stockholm, Sweden

**Keywords:** Anorexia Nervosa, Psychotherapy, Trial, Acceptance and commitment therapy

## Abstract

**Background:**

No specific psychotherapy for adult anorexia nervosa (AN) has shown superior effect. Maintenance factors in AN (over-evaluation of control over eating, weight and shape) were addressed via Acceptance and Commitment Therapy (ACT). The study aimed to compare 19 sessions of ACT with treatment as usual (TAU), after 9 to 12 weeks of daycare, regarding recovery and risk of relapse up to five years.

**Methods:**

Patients with a full, sub-threshold or partial AN diagnosis from an adult eating disorder unit at a hospital were randomized to ACT (*n* = 24) and TAU (*n* = 19). The staff at the hospital, as well as the participants, were unaware of the allocation until the last week of daycare. Primary outcome measures were body mass index (BMI) and specific eating psychopathology. Analyses included mixed model repeated measures and odds ratios.

**Results:**

Groups did not differ regarding recovery and relapse using a metric of BMI and the Eating Disorder Examination Questionnaire (EDE-Q). There were only significant time effects. However, odds ratio indicated that ACT participants were more likely to reach good outcome. The study was underpowered due to unexpected low inflow of patients and high attrition.

**Conclusion:**

Longer treatment, more focus on established perpetuating factors and weight restoration integrated with ACT might improve outcome. Potential pitfalls regarding future trials on AN are discussed. Trial registration number ISRCTN 12106530. Retrospectively registered 08/06/2016.

## Background

Anorexia Nervosa (AN) is a psychiatric disorder with severe and sometimes irreversible medical complications, psychosocial dysfunction and high mortality [1, 2]. In the most recent review, no superior psychotherapy or pharmacological intervention has been documented yet for adults with AN [3]. The evidence base for the treatment of adults with AN is weak. This assertion is supported by previous systematic reviews of randomized controlled trials [4] as well as reviews of the evidence base of treatments for AN (e.g., [5]), regardless of when psychotherapy is provided as the sole treatment or to maintain the outcome of inpatient treatment. Despite intensive efforts and a sizeable number of recent trials (e.g., [6–8]) as well as uncontrolled studies targeting adults with AN (e.g., [9, 10]), no single treatment has consistently emerged as superior to other treatments. Cognitive behavior therapy (CBT), including its latest development (i.e., enhanced CBT), as well as focal psychoanalytic, short-term psychodynamic psychotherapy and interpersonal psychotherapy all have modest support for the treatment of adult AN [11]. There is weak evidence of the efficacy of any psychotropic medications (e.g., [12, 13]). Although hospitalization is recommended by the American Psychiatric Association for adults with AN who are at or below 75 % of their ideal weight [14], remaining cognitive and emotional symptoms after weight gain as well as high rate of relapse during the first 12–18 months after discharge are important reasons for further research on the treatment of adult AN. More research is needed, especially with greater sample sizes, to explore the efficacy of psychotherapy for the treatment of AN. One way to increase sample size is to include those with partial or sub-threshold AN. The severity of ED psychopathology among those with eating disorder not otherwise specified (EDNOS) is similar to those with AN [15]. The implemented changes in the diagnostic criteria of AN from DSM-IV [16] to DSM-5 [17] further validates the inclusion of these patients in trials. Many of these patients would not have been included in previous treatment studies. Now they will receive a full AN diagnosis.

Over-evaluation of weight and shape as well as control over eating and extreme efforts to gain such control are key components in the development and maintenance of AN [18, 19]. Excessive attempts to control internal events (thoughts, feelings, memories and physiological events) maintain psychological problems according to Acceptance and Commitment Therapy (ACT) [20]. As human beings, we persistently attempt to alter the form, frequency or situational sensitivity of private events (i.e., experiential avoidance), even though such behaviors may cause harm [21]. Experiential avoidance includes all escape and avoidance behaviors (overt or covert) that serve this function [22]. In AN, eating may be both precipitated by and followed by thoughts, feelings and body sensations that signal loss of control and being fat, both highly unwanted experiences in AN. Restriction, purging and other compensatory behaviors may then serve as avoidance or escape behaviors of these distressing internal events. The over-evaluation of shape and weight as well as control over eating may be addressed by defusion and acceptance to undermine the (dysfunctional) efforts to modify these internal experiences and, instead, build a flexible behavior repertoire (guided by chosen values) in the presence of these internal events. Prior to initiation of the present study, a case report with ACT for sub-threshold AN (adolescent female with body mass index; BMI > 17.5) was published, showing promising results [23]. Later studies showed that ACT and acceptance-based treatments might be valuable contributions to the treatment of AN. In addition to a case series of three adults (BMI > 18.5) with pre, post and 1-year follow-up [24] as well as an acceptance-based treatment among six adolescents with AN and their caregivers [25], the effect of an ACT-based group treatment on adults with AN [26] and an emotion acceptance behavior therapy trial [27] has been reported. The promising results from these studies further strengthen the assumed potential of ACT for AN and an investigation in a randomized trial.

The aim of the present randomized trial was to compare the effect of ACT with treatment as usual (TAU) after daycare that primarily aimed at discontinuation of starvation. We hypothesized that ACT would result in a higher recovery rate and a reduced risk of relapse compared to TAU among adults with AN after daycare treatment at long-term follow-up.

## Method

### Design

This trial investigated patients who had received 9–12 weeks of daycare at a regional specialist eating disorder unit for adults. The first author generated the randomization sequence (www.randomizer.org) for the allocation of participants to either ACT or TAU. Assessments occurred before daycare, pre ACT/TAU (after daycare), post ACT/TAU and at follow-ups 6, 12, 18 and 24 months post treatment. There was, in addition, a 5-year follow-up (after post treatment; *Mdn* = 5 years, range 2.6–6.3 years). With a hypothesis favoring ACT, a medium effect size, and to obtain a power of at least .85, the study would need 120 participants to detect significant differences between the groups. The recruitment rate was slower than expected and, although the time frame for recruitment was extended, the desired sample size was not reached.

### Participants

Participants included 42 females and one male, with a mean age of *M* = 25.7 (*SD* = 7.15, ranging from 18 to 51 years). Please see Table 1 for demographics. The majority (71 %) lived with a partner, and the rest were singles. Regarding education, 21 % reported having higher education (university), and the rest of the sample had lower education (consisting of compulsory school 12 %, high-school 62 % or vocational school 5 %). The sample’s occupational status was 31 % working, 33.3 % students and the rest not working (9.5 % were unemployed and 26.2 % were on sick leave). Inclusion criteria were ≥18 years of age, AN or EDNOS diagnosis (i.e., sub-threshold or partial AN) prior to daycare and 9–12 weeks completed daycare at the specialized eating disorder unit. BMI was not used as inclusion or exclusion criteria. Partial or sub-threshold AN were defined as fulfilling at least two out of four DSM-IV criteria for AN.

**Table 1:** Demographic characteristics of the participants at pre-assessment (after daycare)

	ACT	TAU	*χ* ^*2*^	*p*	Effect size	OR [95 % CI]
	*N* (%)	*N* (%)			*ω*	
Sex	0 male	1 male^a^				
Diagnosis			0.57	.68	0.12	1.52 [0.49-4.73]
AN	3 (12.5)	4 (21)				
EDNOS	21 (87.5)	15 (79)				
Daycare			0.35	.56	0.09	1.38 [0.44-4.31]
Nine weeks	8 (33.3)	8 (42.1)				
Twelve weeks	16 (66.6)	11 (57.9)				
Marital status			5.11	.024	0.35	3.73 [1.12-12.44]
Single	10 (43.5)	2 (11.1)				
Living together	13 (56.5)	16 (88.9)				
Education			0.42	.71	0.1	1.43 [0.46-4.45]
Lower	18 (75)	15 (83.3)				
Higher	6 (25)	3 (16.7)				
Occupation			3.33	.19	0.28	n.a
Working	10 (41.7)	3 (16.7)				
Student	6 (25)	8 (44.4)				
Not working	8 (33.3)	7 (38.9)				
	*M(SD)*	*M(SD)*	*F*	*p*	Cohen’s *d*	[95 % CI]
Age	26.6(8.1)	24.6(5.8)	0.79	.38	0.29	[-1.81-2.38]
Lowest BMI ever	14.9(2.0)	14.5(2.1)	0.67	.42	0.20	[-0.40-0.80]
Highest BMI ever	21.8(3.4)	23.3(5.0)	3.84	.057	0.37	[-1.59-0.85]
Age first compensate	15.5(2.6)	14.2(5.8)	0.85	.36	0.31	[-0.95-1.57]
Age first diet	14.8(2.7)	13.8(4.6)	0.76	.39	0.28	[-0.79-1.35]

^a^Exclusion of the male participant did not change any comparisons

ACT acceptance and commitment therapy, TAU treatment as usual, AN anorexia nervosa, EDNOS eating disorders not otherwise specified, BMI body mass index

### Procedure

The eating disorder unit approves referral from patients themselves and clinicians from within the region (a county in mid-Sweden with over 340,000 inhabitants) as well as from psychiatric units outside the region. A trained psychiatrist or a trained psychologist examined the patients with the Clinician Version of the Structured Clinical Interview for Axis I Disorders (SCID-CV), combined with the specific ED section from the Structured Clinical Interview Research Version for Axis I Disorders (SCID-I-RV) [28]. The psychiatrist performed a somatic examination, including body weight. The psychologist assessed all these patients using the Eating Disorder Examination (EDE) [29].

**Fig. 1:**
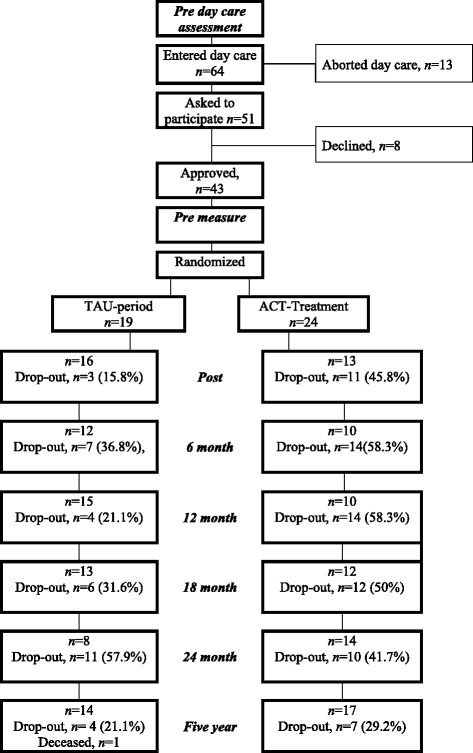
Flow diagram of study participants

All patients diagnosed with full, partial or sub-threshold AN were recommended the daycare program. Only those who entered and participated in the daycare program for nine to twelve weeks were included in the current study. The main aim of the first 9 weeks of the daycare program was to discontinue starvation. Rest and food exposure with support to start eating was the base for daycare*.* The rationale, format, combination of the professional team as well as methods and strategies used within this daycare unit are described in Holmgren et al. [30]. After completing the first nine weeks, an additional and optional 3-week weight gain program was available for all participants before the ACT or TAU treatment started. With the exception of weight gain as a goal, all other aspects of the extended 3-week daycare treatment were the same for the patients. By the seventh week of daycare, patients received information about the present study. In the ninth week, patients who agreed to participate filled out the self-report questionnaires and were subsequently interviewed with the EDE for the second time. After this, in the ninth or twelfth week, the result of the randomization (to ACT or TAU) was disclosed to the participant. The head psychologist at the ED unit was unaware of the allocation of the patients, as sealed envelopes with the allocation information were used. After completion of daycare, the ACT and TAU condition was initiated (see below). Self-report measures and weight were obtained post ACT/TAU and at 6, 12, 18 and 24 months as well as at the 5-year follow-up (please see Fig. 1). Participants were recruited and included between September 2005 and December 2008. The last follow-up assessment took place in May 2013. The ACT treatment was provided at the university clinic. The head psychologist or staff weighed the patients before and after daycare and those randomized to TAU. The psychologist that administered the ACT treatment weighed those randomized to ACT.

At all subsequent follow-ups, participants were called to the daycare clinic to fill out self-report instruments and to be weighed. If the patient was unavailable, the self-report instruments were sent to them and they reported their weight in kilograms using their own scale. Participants who responded to the 5-year follow-up received a cinema voucher for their participation. The regional ethical committee approved the study (Ups 03-519).

### ACT and TAU treatment

The ACT treatment protocol was based on a protocol for the treatment of polysubstance-abusing methadone clients [31]. The sequence and the nature of interventions (e.g., experiential exercises and the use of metaphors, defusion, and acceptance) and the guidance for the therapist were not altered in the current protocol. The proposed common processes that establish and maintain psychopathology among a variety of psychiatric disorders [22] were addressed. The adaptations for the current study protocol included changes in addressing topics to better suit a person struggling with an eating disorder rather than drug use (e.g.,”When did you go on a diet for the first time?” rather than”When did you start using illegal substances?”). When elaborating on experiential avoidance, the therapist may investigate how restriction or purging, rather than use of drugs or alcohol, have worked in the long run for removing or reducing the impact of unpleasant thoughts and feelings. To summarize, the adaptations were changes from substance abuse to eating disorder content in this fashion.

The content and structure of the treatment involved eight obligatory core topics, involving one to four sessions each. The core topics were: 1) Preparing to begin (1-2 sessions), 2) Making contact with the cost and benefits of ED behaviors (e.g., restriction, binging, and purging; 2-3 sessions), 3) Confronting the system: creative hopelessness (1-2 sessions), 4) Excessive emotional control is the problem (1-3 sessions), 5) Introducing experiential willingness (1 session), 6) Distinguishing the person from the programming (1-3 sessions), 7) Experiential willingness (2-3 sessions) and 8) Values and goals (2-4 sessions). The treatment protocol also had four flexible topics from which content could be incorporated into the core topics or be delivered as whole sessions: A) Barriers to emotional acceptance, B) Moving from emotional acceptance to behavior change, C) Accepting responsibility for change and D) Extending emotional willingness into real life: making and keeping commitments. As an example, the core topic 'Values and Goals' would often include content from the flexible topics to promote behavior change.

The ACT treatment consisted of 19 1-h sessions. Three therapists (PhD level or doctoral students, all of them psychologists) administered the treatment. They had extended education and experience of treating patients with other psychiatric disorders with ACT. The senior therapist with extensive experience of treating patients with ED also supervised the two other therapists. Sessions were audio-recorded and audited for supervision and for checking the integrity and quality of the treatment. Nine sessions were randomly selected from 236 audio-recorded sessions and rated for adherence. The manual for adherence rating has been used in a previous study for Obsessive-compulsive disorder [32]. An independent rater, recognized as an expert on ACT treatment, performed the adherence check. The mean rating for overall adherence to the manual was 2.9, and the mean for overall competence of therapist was 3.0 (on a five-point scale, where 1 reflects”not at all”, 3 reflects”somewhat” and 5 reflects”extensively”). According to the adherence-rating manual, a rating of 3 indicates “that the variable occurred several times and was covered at least once in a moderately in-depth manner” [32] (see p. 711).

The TAU condition aimed to support the patients in maintaining regular and sufficient eating as well as to restore weight. Most commonly this was provided by a nurse, but they could also see a physiotherapist, a dietician or a psychologist. Treatment could also target co-morbid disorders. The TAU condition involved any type of further treatment that was available for and chosen by the patients; hence, there was no specific treatment provider. The same was true for the ACT group. However, they were not allowed to receive additional psychotherapy (please see Table 2 for more information regarding use of care). Participants in both conditions were allowed to receive additional daycare during the study period.

**Table 2:** Use of care during the treatment period (pre-post) for both groups

	ACT M ± SD	TAU M ± SD
	N = 22^a^	N = 18^a^
No. of Visits		
Psychiatrist	1.3 ± 1.5	1.8 ± 2.3
Psychologist	15.7 ± 9.2	4.8 ± 4.7
Counselor	0.3 ± 0.8	0.4 ± 1.0
Nurse	0.5 ± 0.2	0.7 ± 3.1
Physiotherapist	0 ± 0	0.3 ± 1.2
Care assistant	4.0 ± 4.3	6.3 ± 5.0
Dietician	0 ± 0	0 ± 0
Psychiatric emergency	0.5 ± 0.2	0.6 ± 0.2
No. of days in Care		
Daycare	10.6 ± 15.7	18.8 ± 22.6
Inpatient care	1.7 ± 7.5	1.7 ± 7.1

^a^Two participants in the ACT group and one in the TAU group did not consent to use data from their psychiatric records

### Measures

#### Interviews

The SCID-I-RV [28] was used prior to daycare to assess ED. As stated previously, either a trained psychiatrist (third author) or a trained clinical psychologist examined the patients. The overall Kappa coefficient between the psychiatrist and the psychologist was .79 for categorical axis I diagnoses, based on four randomly selected SCID-I interviews. The EDE [29] was used to assess ED prior to and after daycare. This semi-structured interview is considered the gold standard for assessment of ED. As in previous research [33], a short version of the EDE was used in the present study to investigate ED diagnoses as operationalized by the interview. The clinical psychologist at the daycare clinic, who had received training in the procedure by the senior author, performed the EDE interview.

### Self-report measures

Self-report measures were used at pre and post ACT/TAU and on subsequent follow-up occasions. The Eating Disorder Examination Questionnaire (EDE-Q) [34] is a self-report version of the EDE and provides a global score and four sub-scales: Restraint, Eating Concern, Shape Concern and Weight Concern. The Montgomery Åsberg Depression Rating Scale (MADRS-S) [35] assessed depressive symptomatology. The Quality of Life Inventory (QOLI) [36] measured the satisfaction with different areas in life. The Perceived Social Support (PSS) [37] assessed perceived social support from friends and from family. Six sub-scales of the Symptom Check List (SCL-90; Somatization, Obsessive-Compulsive, Interpersonal sensitivity, Anxiety, Anger-Hostility and Phobic Anxiety, from which the Global Severity Index was estimated) assessed current psychological symptoms [38, 39]. The Rosenberg Self-Esteem Scale (RSE) [40] assessed global self-esteem. The Body Shape Questionnaire (BSQ) [41] asked for concerns about body shape. The Ways of Coping Questionnaire (WCQ) [42] identified thoughts and actions used to cope with a specific stressor. Finally, the sub-scales Drive for thinness, Bulimia, Body satisfaction, Ineffectiveness, Interpersonal distrust and Interoceptive awareness from the Eating Disorder Inventory-2 (EDI) [43] assessed attitudes, feelings and behaviors associated with eating disorders. The 5-year follow-up included only the EDE-Q, BMI and the Clinical Impairment Assessment scale (CIA) [44] that assessed the impact of eating disorders on psychosocial functioning. This was done to increase the likelihood of participation. During the pre-assessment, Cronbach’s alphas ranged between .71 and .97 for the instruments above. At the 5-year follow-up, the Cronbach’s *α* for the CIA was = .98. The EDE-Q and BMI were the primary outcome variables. All other measures were secondary outcome variables.

Lower scores indicate less symptoms on the EDE-Q, MADRS-S, EDI-2, CIA, SCL-90 and the BSQ. Higher scores indicate higher quality of life (QOLI), more use of coping strategies (WCQ), more perceived social support from family and friends (PSS) and lower self-esteem on the RSE.

### Use of health care

Use of health care was recorded from the patients' psychiatric records and was summarized from pre to post ACT / TAU, and from post through 12 months follow-up. The number of visits to any psychiatrist, psychologist, counselor, nurse, physiotherapist, care assistant or dietician was recorded. Additional daycare usage at the unit as well as other forms of psychiatric daycare were recorded as number of days for each service, respectively. Visits to the psychiatric emergency unit were coded in terms of number of visits, and inpatient care was recorded as number of days.

We did not sytematically ask for adverse events or harms

### Statistical analysis

For nominal data, Pearson’s *χ*^*2*^ or Fishers exact test and odds-ratios were calculated. Effect sizes for nominal data are presented in terms of *ω,* where 0.1, 0.3 and 0.5 are used as guidelines for small, medium and large effect sizes. Mann–Whitney *U* test was used to analyze the groups’ use of health care due to violation of homogeneity of variances. For between-group comparisons at pre ACT/TAU, one-way ANOVAs were used. Cohen’s *d* was used as effect size measure, where 0.2 is considered small, 0.5 medium and 0.8 large [45]. A mixed model repeated measures (MMRM) analysis was used to investigate effects using an intent-to-treat sample [46], with two between levels (ACT and TAU), six or seven within levels (EDE-Q and BMI were recorded seven times) and one covariate (the pre-measure for each dependent variable). Mixed regression models use all available data from all subjects, which makes it a suitable approach for intent-to-treat analysis. The analyses were conducted using an unstructured covariance structure, followed by compound symmetry, Toeplitz, and the heterogeneous compound symmetry covariance structures. The model with the fewest parameters is reported unless another model had significantly better model fit, as determined by comparison of the restricted log-likelihood. Effect sizes (Cohen’s *d*) for F values were based on suggestions for repeated measures and multilevel designs [47, 48], and effect sizes for planned contrasts were used to dissemble interactions [49]. Based on Swedish norms [50], a global EDE-Q score above +1 *SD* from the general population mean (2.83) was used as the clinical cut-off for ED psychopathology. The criterion for clinical significance regarding weight was a BMI of at least 19. As a cut-off to identify whether participants reached good outcome or not, both a BMI of at least 19 and an EDE-Q global score at or below 2.83 was required. If one of these measures did not reach the criteria, the participant did not reach good outcome. SPSS version 22 [51] was used for all analyses.

## Results

### Pre daycare assessment

Prior to daycare, there was no significant difference between the groups' BMI (ACT, *M* = 16.8, *SD* = 2.3, and TAU *M* = 17.1, *SD* = 2.73; *F*(1,41) = 0.12, *p* = .73, *d* = -0.12 [95 % CI: -0.86-0.63]) or diagnosis as assessed with the EDE interview (*χ*^*2*^ = 0.22, *p* = .71, OR = 1.29 [95 % CI: 0.42-4.02]).

### Pre ACT/TAU (after daycare): Demographics, diagnosis and self-report measures

Prior to the ACT/TAU treatment, there was a significant difference in marital status between the groups, with a larger portion of singles in the ACT group compared to the TAU group (see Table 1). There were no significant differences between the groups regarding level of education, occupation, age, highest or lowest BMI ever or age when the participant started to use compensatory behaviors or go on a diet. The groups showed no significant difference regarding diagnosis as operationalized according to the EDE interview at pre ACT/TAU (i.e., after nine weeks of daycare) or the use of nine or twelve weeks of daycare.

There were no significant differences between the groups' BMI (*F*(1,41) = 0.58, *p* = .45), the EDE-Q global score or its sub-scales (0.7 < *F* < 2.3, .14 < *p* < .41), or EDI sub-scales (0.003 < *F* < 0.79, .38 < *p* < .96; please see Table 3). No significant differences were found on the sub-scales of the SCL, WCQ, PSS, BSQ, MADRS-S, QOLI or the RSE (*F* < 0.99, *p* > .055). The BMI range for the TAU group was 14.47-23.95, and the range for the ACT group was 13.21-20.90.

**Table 3:** Means (standard deviations) for the outcome measures at pre-assessment for the ACT and TAU group

	ACT *n* = 24	TAU *n* = 19
	*M (SD)*	*M (SD)*
BMI	17.5 (2.3)	18.1 (2.6)
EDE-Q	3.1 (1.3)	3.6 (1.3)
Restraint	2.4 (1.4)	3.1 (1.7)
Eating	2.9 (1.1)	3.2 (1.2)
Shape	3.8 (1.6)	4.4 (1.4)
Weight	3.2 (1.5)	3.6 (1.3)
BSQ	116.0 (37.2)	118.4 (35.6)
EDI		
D	10.6 (6.2)	11.7 (5.8)
B	1.8 (4.0)	1.4 (3.2)
Bd	16.4 (7.8)	15.3 (6.8)
Inef	9.6 (6.5)	11.4 (6.6)
Perf	5.7 (3.5)	6.7 (5.2)
Interp	3.6 (4.0)	3.5 (3.9)
Intero	7.7 (6.6)	9.6 (7.1)
MADRS	19.9 (11.8)	21.7 (10.3)
QOLI	1.1 (1.4)	0.1 (1.8)
RSE	1.7 (1.8)	1.8 (1.8)

*BMI* body mass index, *EDE-Q* eating disorder examination questionnaire, *BSQ* body shape questionnaire, *EDI* eating disorder inventory (*D* drive for thinness, *B* bulimia, *Bd* body dissatisfaction, *Inef* ineffectiveness, *Perf* perfectionism, *Interp* interpersonal distrust, *Intero* interoceptive awareness), *MADRS-S* montgomery asberg depression rating scale self-report, *QOLI* quality of life inventory, *RSE* rosenberg self-esteem

### Use of health care during the ACT/TAU treatment period

Although the TAU group had fewer visits to psychologists compared with the ACT group (*U* = 357, *z* = 3.59, *p* < .001, *n* = 42), both groups consumed an equal amount of care in terms of visits to psychiatrist, counselor, nurse, physiotherapist, care assistant, dietician, days of additional daycare/other psychiatric daycare, visits to psychiatric emergency unit, inpatient care as well as total consumed care, including visits to psychologists (0.42 > *z* > -1.52, 1 > *p* > .13).

### Attrition from study and drop-out from ACT treatment

At post and 12 months, there was a significant difference in attrition between the two groups (*χ*^2^ = 4.36 and 6.06, *p* = .037 and .014 respectively; please see Fig. 1).

Out of the 24 randomized participants in the ACT group, 14 (58.3 %) were considered ACT treatment completers as they attended at least 16 sessions; four participants aborted treatment before it began, two participants attended two and three sessions respectively (were highly ambivalent from the onset), two participants attended five sessions (one needed hospitalization due to AN-unrelated somatic illness) and two participants attended 12 and 13 sessions respectively (one started another treatment for which the participant had been on a wait list). The ACT treatment completers were more inclined to attend to post and follow-up measurements than those who dropped out. One participant in the TAU condition completed suicide.

### Missing data

During post-measurement, there were no significant differences between those missing versus those complying concerning level of depression, anxiety or eating disorder symptoms. However, those who were missing at post in the TAU group (3 participants) had significantly higher BMI at pre-daycare and at the pre-assessment compared to those not missing at post (*F* > 5.38, *p* < .033). Those missing at post in the ACT group (11 participants) showed significantly higher levels of depression (MADRS; *F*(1,22) = 7.42, *p* = .013) compared with those not missing at post.

### Outcome: Clinical significance

During the pre-assessment (end of daycare), three participants in the ACT group and zero participants in the TAU group had reached the criteria for good outcome based on the combined criteria (BMI ≥ 19 and EDE-Q global score ≤ 2.83). The ACT and the TAU group were compared regarding the number of participants with good outcome, excluding the three ACT participants who had reached good outcome at pre-assessment. During post and on all subsequent follow-ups, there was no significant difference between groups (2.06 < *χ*^*2*^ < 0.19, .15 < *p* < .66). Notably, those who received ACT were non-significantly more likely to reach good outcome (i.e., BMI ≥ 19 and EDE-Q global score ≤ 2.83) than participants in the TAU group at post; OR = 2.50 [95 % CI: 0.3-16.1], where 4 of 16 in the ACT group and 2 of 17 in TAU group reached good outcome. At 6 months, OR = 1.50 [95 % CI: 0.2-9.4], where 3 of 11 in the ACT group and 3 of 15 in the TAU group reached good outcome. At 18 months, OR = 1.61 [95 % CI: 0.3-8.3], where 5 of 12 in the ACT group and 4 of 13 in the TAU group reached good outcome. At 24 months, OR = 5.00 [95 % CI: 0.5-51.8], where 5 of 14 in the ACT group and 1 of 9 in the TAU group reached good outcome. Finally, at the 5-year follow-up, OR = 2.70 [95 % CI: 0.6-12.2], where 9 of 16 in the ACT group and 5 of 14 in the TAU group reached good outcome. However, at 12 months, OR = 0.69 [95 % CI: 0.1-3.6], where 3 of 11 in the ACT group and 6 of 17 in the TAU group reached good outcome.

### Outcome from pre to 5-year follow-up on the BMI and the EDE-Q

**Fig. 2:**
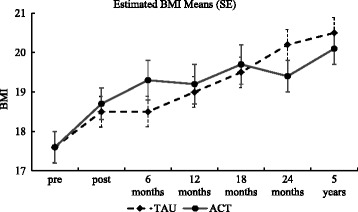
Estimated BMI means (bars representing standard error: SE) at all assessments

The 5-year follow-up included only BMI, EDE-Q and the CIA. The results for the BMI and the EDE-Q are presented first. There was a significant main effect for time but no main effect for group or interaction regarding BMI (please see Table 4 for results from the MMRM analysis and Fig. 2 for an illustration of the estimated means for the BMI across time). Time effects are presented in terms of contrasts from pre to post, from pre to 6 months and so on within each group (mean estimates and SE are presented in Table 5). The time effect was due to significant increase in BMI for the ACT group from pre to post (*d* = 0.59), pre to 6 months (*d* = 0.78), to 12 months (*d* = 0.70), to 18 months (*d* = 0.87), to 24 months (*d* = 0.70) and from pre to five years (*d* = 0.99; please see Table 5). For the TAU group, from pre to 12 months (*d* = 0.61), pre to 18 months (*d* = 0.79), to 24 months (*d* = 1.02) and from pre to five years (*d* = 1.12).

**Table 4:** Group, time and interaction effects from the MMRM analysis

	Group effect		Time effect		Interaction effect	
	*F (df)*	*p*	*F (df)*	*p*	*F (df)*	*p*
BMI^a^	0.07 (1,47.8)	.93	7.12 (6,74.8)	<.001	0.61 (6,74.9)	.72
EDE-Q^a^ Global	0.12 (1,51.44)	.73	9.09 (6,48.3)	<.001	0.62 (6,48.3)	.71
Restraint	0.18 (1,52.2)	.67	1.80 (6,61.7)	.11	0.25 (6,61.8)	.96
Eating	0.02 (1,46.8)	.89	14.83 (6,60.7)	<.001	0.33 (6,60.8)	.92
Shape	0.14 (1,53.2)	.71	9.68 (6,44.6)	<.001	1.16 (6,44.6)	.34
Weight	0.10 (1,55.2)	.76	5.40 (6,64.5)	<.001	0.67 (6,64.6)	.67
BSQ	0.74 (1,60.7)	.40	5.67 (5,63.3)	<.001	0.39 (5,63.3)	.86
EDI						
D	0.92 (1,61.1)	.76	3.62 (5,45.7)	.008	0.39 (5,45.7)	.85
B	0.15 (1,43.1)	.70	0.68 (5,63.2)	.64	0.81 (5,63.1)	.54
Bd	3.60 (1,53.0)	.063	3.75 (5,71.1)	.005	0.81 (5,71.1)	.54
Inef	2.37 (1,58.2)	.13	6.23 (5,63.3)	<.001	0.73 (5,63.3)	.61
Perf	0.25 (1,62.0)	.62	0.63 (5,60.8)	.68	1.53 (5,60.7)	.19
Interp	0.003 (1,51.6)	.96	1.88 (5,65.0)	.11	1.52 (5,65.0)	.20
Intero	0.68 (1,63.5)	.41	2.37 (5,54.8)	.051	1.38 (5,55.8)	.24
MADRS	3.91 (1,45.1)	.054	2.14 (5,125.6)	.064	1.33 (5,125.5)	.26
QOLI	0.11 (1,59.0)	.74	5.23 (5,41.6)	.001	0.38 (5,41.5)	.86
RSE	0.06 (1,49.1)	.82	6.08 (5,81.1)	<.001	0.88 (5,81.0)	.50
WCQ	0.02 (1,38.2)	.88	0.95 (5,118.6)	.45	1.78 (5,118.8)	.12
SCL GSI	0.90 (1,56.6)	.35	1.15 (5,55.1)	.34	0.64 (5,55.2)	.67
PSS fa	0.29 (1,55.2)	.59	1.32 (5,63.1)	.27	0.70 (5,63.1)	.63
PSS fr	0.30 (1,38.6)	.59	0.63 (5,123.1)	.67	0.10 (5,123.1)	.99

^a^primary outcome variables were followed up at post, 6, 12, 18, 24 months and five years, and the secondary variables were followed up at 24 months. The pre-measure for each outcome variable was used as covariate. ACT *n* = 24, TAU *n* = 19

*MMRM* mixed model repeated measures, *BMI* body mass index, *EDE-Q* eating disorder examination questionnaire, *BSQ* body shape questionnaire, *EDI* eating disorder inventory (*d* drive for thinness, *b* bulimia, *Bd* body dissatisfaction, *Inef* ineffectiveness, *Perf* perfectionism, *interp* interpersonal distrust, *Intero* interoceptive awareness), *MADRS-S* montgomery asberg depression rating scale self-report, *QOLI* quality of life inventory, *RSE* rosenberg self-esteem, *WCQ* ways of coping questionnaire, *SCL GSI* symptom checklist global severity index, *PSS* perceived Social Support (fa) family and (fr) friends

**Table 5:** Estimated means (standard errors) for the TAU and the ACT group at all assessments

	pre		post		6 months		12 months		18 months		24 months		5 year	
	TAU	ACT	TAU	ACT	TAU	ACT	TAU	ACT	TAU	ACT	TAU	ACT	TAU	ACT
	*M(SE)*	*M(SE)*	*M(SE)*	*M(SE)*	*M(SE)*	*M(SE)*	*M(SE)*	*M(SE)*	*M(SE)*	*M(SE)*	*M(SE)*	*M(SE)*	*M(SE)*	*M(SE)*
BMI	17.6(0.4)	17.6(0.4)	18.5(0.5)	18.7(0.4)^*^	18.5(0.5)	19.3(0.5)^**^	19.0(0.5)^*^	19.2(0.5)^**^	19.5(0.5)^**^	19.7(0.5)^**^	20.2(0.6)^***^	19.4(0.4)^**^	20.5(0.5)^***^	20.1(0.4)^***^
EDE-Q	3.3(0.3)	3.1(0.2)	2.2(0.3)^†^	2.1(0.3)^***^	2.2(0.3)^**^	2.2(0.3)^**^	1.4(0.3)^***^	2.0(0.3)^**^	2.3(0.3)^*^	2.4(0.3)^*^	2.5(0.4)^*^	2.4(0.3)^*^	1.9(0.3)^**^	2.3(0.3)^*^
Restraint	2.7(0.3)	2.5(0.3)	1.9(0.4)	2.0(0.4)	1.7(0.4)	2.0(0.4)	2.1(0.4)	2.4(0.4)	1.9(0.4)	2.0(0.4)	2.0(0.5)	2.4(0.4)	2.0(0.4)	2.3(0.4)
Eating	3.0(0.3)	3.0(0.2)	1.8(0.3)^***^	1.5(0.3)^†^	1.8(0.3)^†^	1.7(0.3)^**^	0.8(0.3)^***^	1.0(0.3)^***^	1.9(0.3)^**^	1.9(0.3)^**^	1.6(0.3)^**^	1.7(0.3)^**^	1.5(0.3)^***^	1.8(0.3)^**^
Shape	4.0(0.3)	3.9(0.3)	3.1(0.3)^**^	2.8(0.4)^**^	3.2(0.4)	3.1(0.4)	1.3(0.4)^***^	2.4(0.4)^**^	3.1(0.4)^*^	3.3(0.4)	3.5(0.4)	3.2(0.4)	2.5(0.4)^**^	2.8(0.3)^*^
Weight	3.3(0.3)	3.2(0.3)	2.2(0.3)^**^	2.1(0.3)^**^	2.4(0.4)^*^	2.0(0.4)^**^	1.6(0.3)^***^	2.1(0.4)^*^	2.4(0.4)^*^	2.1(0.4)^**^	2.6(0.4)	2.2(0.3)^**^	1.8(0.3)^*^	2.0(0.3)^**^
BSQ	114.6(5.9)	114.4(5.2)	100.7(6.1)^**^	91.0(6.2)^†^	100.9(6.6)	94.3(7.0)^**^	100.0(6.3)	94.4(7.1)^*^	100.4(6.6)	97.9(6.9)	101.2(7.3)	94.6(6.4)^*^		
EDI														
D	10.8(1.0)	10.6(0.9)	8.7(1.0)^*^	7.8(1.1)^**^	8.0(1.1)^*^	8.4(1.2)	7.8(1.1)^*^	7.8(1.2)	7.6(1.1)	7.4(1.2)^*^	8.8(1.3)	7.8(1.1)		
B	1.2(0.4)	1.2(0.4)	0.6(0.4)	1.3(0.5)	1.9(0.5)	1.4(0.5)	1.5(0.4)	0.9(0.5)	1.9(0.5)	1.7(0.5)	1.5(0.6)	1.3(0.5)		
Bd	15.2(1.1)	15.3(1.0)	13.6(1.2)	10.5(1.2)^†^	14.3(1.3)	12.0(1.4)^*^	13.4(1.2)	11.2(1.4)^**^	13.7(1.3)	11.4(1.3)^*^	13.7(1.4)	10.8(1.2)^*^		
Inef	10.5(1.1)	10.1(1.0)	5.3(1.2)^***^	7.4(1.3)^*^	6.1(1.3)^**^	7.8(1.4)	7.2(1.2)^*^	10.2(1.4)	5.9(1.3)^**^	8.0(1.4)	7.2(1.5)	9.2(1.3)		
Perf	5.9(0.7)	5.7(0.6)	5.2(0.7)	6.7(0.7)	5.7(0.8)	6.5(0.8)	6.0(0.7)	5.4(0.8)	5.2(0.8)	4.9(0.8)	4.7(0.8)	5.5(0.7)		
Interp	3.1(0.5)	3.1(0.4)	2.0(1.5)	3.0(0.5)	1.9(0.5)	2.5(0.5)	2.7(0.5)	2.0(0.6)	2.4(0.5)	1.7(0.5)	2.0(0.6)	1.7(0.5)		
Intero	8.6(1.1)	8.3(1.0)	4.7(1.2)	7.3(1.2)	6.1(1.3)	6.1(1.4)	6.7(1.2)	5.7(1.4)	5.9(1.3)	6.2(1.3)	4.2(1.4)	7.4(1.2)		
MADRS	20.1(2.0)	19.5(1.8)	12.4(2.1)	17.3(2.3)	15.1(2.4)	18.7(2.6)	12.5(2.2)	20.9(2.6)	13.4(2.3)	17.7(2.4)	13.7(2.8)	17.8(2.2)		
QOLI	0.5(0.3)	0.8(0.3)	1.7(0.3)^***^	1.6(0.4)^**^	1.5(0.4)^**^	1.2(0.4)	1.5(0.4)^*^	1.1(0.4)	1.4(0.4)	1.3(0.4)	1.1(0.4)	0.8(0.4)		
RSE	1.8(0.4)	1.8(0.3)	3.0(0.4)^**^	3.4(0.4)^***^	3.0(0.4)^**^	3.3(0.5)^**^	2.5(0.4)	2.8(0.5)^*^	3.1(0.4)^*^	2.4(0.5)	2.9(0.5)	2.3(0.4)		

*BMI* body mass index, *EDE-Q* eating disorder examination questionnaire, *BSQ* body shape questionnaire, *EDI* eating disorder inventory (*d* drive for thinness, *b* bulimia, *Bd* body dissatisfaction, *Inef* ineffectiveness, \*Perf* perfectionism, *interp* interpersonal distrust, *Intero* interoceptive awareness), *MADRS-S* montgomery asberg depression rating scale self-report, *QOLI* quality of life inventory, *RSE* rosenberg self-esteem

*p < .05, **p < .01, ***p < .001 significant time effect within each group from pre-measure

There were significant main effects for time on the EDE-Q Global score concerning the Eating, Shape and Weight concern sub-scales but no group or interaction effects (see Table 4). The significant time effect on the EDE-Q global score was due to significant decrease (less ED symptoms) for both groups from pre to subsequent follow-ups up to five years (ACT group *d* = 1.02, 0.74, 0.85, 0.52, 0.54, 0.56; TAU group *d* = 0.98, 0.83, 1.37, 0.63, 0.57, 0.81; please see Table 5).

The significant time effect on the EDE-Q Eating concern scale was due to significant decrease (less eating concerns) for both groups from pre to all subsequent follow-ups up to five years (ACT group *d* = 1.32, 0.96, 1.40, 0.71, 0.81, 0.70; TAU group *d* = 1.11, 0.98, 1.68, 0.76, 0.92, 0.93).

The significant time effect on the EDE-Q Weight concern scale was due to significant decrease (less weight concerns) for the ACT group from pre to all subsequent follow-ups up to five years (*d* = 0.81, 0.78, 0.70, 0.67, 0.67, 0.73) and on all follow-ups except pre to 24 months for the TAU group (*d* = 0.76, 0.57, 1.03, 0.55, n.s., 0.89).

The significant time effect on the EDE-Q Shape concern scale was due to significant decrease (less shape concerns) from pre to post (*d* = 0.78), pre to 6 months (*d* = 0.46), pre to 12 months (*d* = 0.85) and from pre to 5-year follow-up (*d* = 0.63) in the ACT group and significant decrease from pre to post (*d* = 0.67), from pre to 12 and 18 months (*d* = 1.58 and 0.53) and from pre to 5-year follow-up (*d* = 0.85) in the TAU group.

The CIA, administered only at the 5-year follow-up, showed no significant difference (*t*(29) = 0.42, *p* = .68, d = 0.15 [95 % CI: -5.23-5.53]) between the TAU (*M* = 17.21, *SD* = 14.15, *n =* 14) and the ACT group (*M* =14.88, *SD* = 16.15, *n* = 17).

### Outcome from pre to 24 months for the remaining self-report scales

The remaining measures were followed up from pre to 24 months and are presented below. The EDI showed significant time effects on the Drive for thinness, Body dissatisfaction and the Ineffectiveness sub-scales (Table 4). No other significant main or interaction effects were found. On the Drive for thinness scale, the significant time effect was due to the ACT group mean estimate of change (less drive for thinness) from pre to post (d = 0.79) and from pre to 18 months (d = 0.47), and the TAU group mean estimate of change (less drive for thinness) from pre to post and up to 18 months (d = 0.58, 0.54, 0.51, 0.46; see Table 5). The significant time effect on the Body dissatisfaction sub-scale of the EDI was due to the ACT group mean estimate of change (less body dissatisfaction) from pre to post and on all subsequent follow-ups up to 24 months (*d* = 1.05, 0.61, 0.70, 0.62, 0.60) and no significant contrasts for the TAU group. The significant time effect on the Ineffectiveness sub-scale of the EDI was due to the ACT group mean estimate of change (less ineffectiveness) from pre to post (d = 0.57), and the TAU group mean estimate of change (less ineffectiveness) from pre to post, 6, 12 and 18 month follow-ups (d = 1.11, 0.80, 0.54, 0.68).

There was a significant effect of time on the BSQ (see Table 4) due to the ACT group mean estimate of change (less body shape concerns) from pre to post up to 12 months (d = 1.13, 0.72, 0.64) and at 24 months (d = 0.46) and due to the TAU group mean estimate of change (less body shape concerns) from pre to post (d = 0.67).

There was a significant time effect, indicating a higher quality of life, on the QOLI (see Table 4) due to the ACT group mean estimate of change from pre to post (*d* = 0.69) and from pre to post, 6 months, and pre to 12 months in the TAU group (*d* = 1.05, 0.69, 0.57: please see Table 5).

There was a significant time effect, indicating lower self-esteem, on the RSE (please see Table 4) due to the ACT group mean estimate of change from pre to post, to 6 months and to 12 months (*d* = 0.95, 0.84, 0.58) and from pre to post and pre to 6 months (*d* = 0.74 and 0.71) as well as pre to 18 months in the TAU group (*d* = 0.59; please see Table 5). No other significant effects were found on the self-report scales, see Table 4.

### Use of health care from post-treatment up to 12-month follow-up

From post treatment to 6 months follow-up, the ACT group had significantly fewer visits to psychiatrists (*U* = 111, *z* = -2.4, *p* = .016, *n* = 39) and less daycare utilization (*U* = 143, *z* = -2.06, *p* = .039, *n* = 39). No other significant differences were found (-1.66 < *z* < 1.33, .096 < *p* < .89) regarding use of care during post to 6 months follow-up. From 6 to 12 months follow-up, there was no significant difference between the groups’ use of care (-1.8 < *z* < 1.24, .071 < *p* < .87). From post to 12-month follow-up, both groups showed equal amount of care use (*U* = 109.5, *z* = - 1.76, *p* = .079) as well as visits (*U* = 97.5, *z* = -1.76, *p* = .078) over all categories.

During the first year after the treatment, there was a significant difference regarding visits to psychiatrist between ACT treatment completers (*M* = 0.75, *Mdn* = 0) versus the TAU group (*M* = 2.0, *Mdn* = 1; *U* = 58, *z* = -2.57, *p* = .01, *n* = 31). There was no significant difference regarding visits to psychologists between ACT treatment completers (*M* = 1.08, *Mdn* = 0) and TAU participants (*M* = 4.13, *Mdn* = 1; *p* = .16).

## Discussion

The purpose of the current study was to compare the effect of ACT with treatment as usual (TAU) after daycare. The results indicated no significant differences between the groups in terms of good outcome (a combination of a BMI ≥ 19 and EDE-Q global score ≤ 2.83). Odds-ratio analyses indicated that the ACT group was more likely to reach good outcome at all follow-ups except at 12 months. However, the confidence intervals show that these ratios are not statistically significant, and there is a risk that the ACT participants are a selected group (due to the attrition) compared with the TAU group.

The MMRM analyses on the primary variables BMI and the EDE-Q showed no group or interaction effects. However, significant improvements across time were found on BMI and the EDE-Q global score (the eating, weight and the shape concern scales). The increase in BMI was evident at all assessment points in the ACT group, with medium to large effect sizes (ES). In the TAU group, all assessment points except post and 6 months showed significant improvement (medium to large ES). The EDE-Q global score showed significantly less ED symptoms at all assessments for both groups, with medium to large ES. The primary variables in the present study showed very little difference between the two groups. A noteworthy distinction is that the ACT group seemed to gain weight somewhat faster than the TAU group at post and at 6 months. However, there were medium to large effect sizes within both groups at nearly all contrasts as measured from pre to the 5-year assessment.

The secondary variables yielded no group or interaction effect. The eating disorder inventory scales, body shape and quality of life showed significant improvements across time. There was a significant deterioration across time regarding self-esteem. At the 24-month assessment (the endpoint for the secondary measures), there were only significant contrasts within the ACT group. These were improvements on the BSQ (small ES) and the Body dissatisfaction scale (medium ES) of the EDI-2.

The TAU group visited psychiatrists more often than the ACT treatment completers from post to 12-month follow-up. Speculatively, the structured and weekly ACT treatment reduced the subjective need for psychiatrist visits compared with the less structured and occasional treatment condition in TAU condition.

The current study is not maintenance treatment or relapse prevention after inpatient care. Nor is it easily defined as acute treatment due to the relatively short daycare that preceded the ACT or TAU treatment periods as well as the nature of the daycare's aim to expose the patients to eating and not primarily focusing on weight gain. However, since the current study is most similar to prior acute studies, comparisons are made with acute treatment trials and uncontrolled studies. Fairburn and colleagues [9] investigated the effect of enhanced CBT for two samples with BMI < 17.5 from pre to 60 weeks. At the pre-assessment and at 60-weeks follow-up for the intent-to-treat sample, the BMI was 16.1 (*SD* = 1.2) and 17.8 (2.0), respectively. In the present study, those with a BMI < 17.5 (TAU; *n* = 11 and ACT; *n* = 12) recorded a BMI of 15.1 (1.5) prior to daycare, and BMI = 17.8 (3.0) at 12 months. The high drop-out of participants in the current study limits any direct comparisons with Fairburn’s study. However, the participants in the current study show a similar increase and status in BMI, as do the participants in Fairburn’s study.

The daycare treatment plus the ACT or TAU treatments in the current study were comparably effective in significant weight gain and in decreasing ED symptomatology. However, the daycare is a more intensive and expensive treatment, and that should be kept in mind when these treatments are compared to each other.

The psychotherapy delivered in this study (19 weekly sessions) was relatively brief compared to outpatient trials, with 40 sessions over 10 months [8] or 25 sessions extended over a year [52]. Also, Fairburn proposed and investigated [9, 18] a 40-session protocol for those with a BMI < 17.5. Similarly, a prolonged treatment when using ACT for AN might be needed in order to be effective. Furthermore, the use of a weight criterion for inclusion after daycare or hospitalization [53, 54] might provide a firmer foundation for the full benefit of any psychotherapy. However, preliminary data suggest that patients with a BMI below 15 can benefit from enhanced CBT for ED [9]. The present study included patients after daycare without requiring weight gain for inclusion, and there was no modification of the daycare treatment in any way, which lends support to high external validity of the study to the context in which it was carried out. The theoretical argument (undermining the control agenda) for investigating the efficacy of ACT for AN might still be valid; although, a longer treatment plus a BMI above 15 might be a sounder starting point. In addition, ensuring a high level of therapeutic skills and higher adherence to the treatment manual are crucial for the further investigation of ACT for AN. Finally, the ACT treatment protocol used in this study was delivered with a general approach to psychopathology. Further investigations using ACT for ED need to focus more on weight restoration and other known ED maintenance factors.

Attrition from the ACT group was significantly higher at post but not cumulatively at 6 and 12 months follow-up compared with the TAU group. A large number of patients in the ACT group did not complete the treatment. The majority dropped out at an early phase and clearly indicated their ambivalence to proceed with treatment in the first or second session. In the current study, 58 % of the ACT participants completed the treatment. In Fairburn et al.’s study, 64 % completed the treatment of 40 individual sessions [9]. The more intensive daycare (9 or 12 weeks) prior to ACT might have refrained some patients from further treatment. Some patients expressed discontent regarding the heavy burden of self-report questionnaires included in the study, which probably contributed to the high attrition rate. In the design of the study, attrition was anticipated. The head psychologist at the daycare unit, who was not a therapist in the study, contacted participants for follow-up assessments given his strong and well-known therapeutic relationship with the patients. In spite of this, and speculatively due to the high load of measurements in addition to the well-known ambivalence of the patient-group toward treatment, attrition was high. However, attrition was higher in ACT at post measurement compared to TAU for unknown reasons. Future studies should investigate predictors of drop-out in general and in relation to specific treatments.

Several limitations need to be addressed. There was an unexpected slow recruitment rate to the study and, despite extending the recruitment period, the study is short of power. Furthermore, the attrition was high, severely limiting the possibility of drawing valid conclusions from the outcome, with the exception of data from the 5-year follow-up. The length of each visit (number of contact hours) was not recorded in the psychiatric records. However, the length of each visit was fairly fixed within each category of caregiver, and the analysis showed no significant differences regarding number of visits to care givers between the groups. At the onset of the study, psychometrically sound and relevant process measures in Swedish were absent. Due to that, processes targeted in ACT were not investigated. The shortcomings in the present randomized trial (not reaching desired sample size and high drop-out rate) have been encountered in other studies, and it is acknowledged that evaluating the effectiveness of treatment for AN with this design is difficult [55].

Since there is no superior treatment for AN and the majority of trials have concluded equal effects as compared with their control treatments [3, 4], one could question the utility of the current conceptualization of AN focusing on over-evaluation of shape, weight and control over eating. Compelling arguments for a perspective shift have been aired, suggesting a focus on genetics, intestinal microbiota, inflammatory processes as well as neuroscience and their interactions with psychosocial factors [56]. Another argument is to view most of the psychopathology of AN as a consequence of emaciation [57] to advance the understanding and treatment of AN. However, until such evidence has emerged, focus on outcome variables combining biological and psychological variables (e.g., BMI and over-evaluation of shape and weight) remains a plausible approach.

Further studies using ACT for full, sub-threshold or partial AN should consider investigating the effect of a more intensive and prolonged treatment for patients with a BMI of at least 15 and focus more on ED specific pathogenic maintenance factors. Finally, in future studies, the investigators should balance the number of self-report measures with the need to assess and detect clinically significant changes to avoid assessment overload.

## Conclusions

The current study found no significant difference in outcome between the TAU and the ACT treatment regarding recovery and relapse. However, as in previous studies, significant improvements on weight gain and ED symptomatology were found in both groups.

## Abbreviations

ACT, acceptance and commitment therapy; AN, anorexia nervosa; BMI, body mass index; BSQ, body shape questionnaire; CBT, cognitive behavior therapy; CIA, clinical impairment assessment scale; DSM, diagnostic and statistical manual of mental disorders; ED, eating disorder; EDE, eating disorder examination; EDE-Q, eating disorder examination questionnaire; EDI, eating disorder inventory; EDNOS, eating disorder not otherwise specified; ES, effect size; MADRS-S, montgomery asberg depression rating scale – self rating; MMRM, mixed model repeated measures; PSS, perceived social support; QOLI, quality of life inventory; RSE, rosenberg self-esteem scale; SCID-CV, structured clinical interview for axis i disorders- clinical version; SCID-I-RV, structured clinical interview for axis i disorders – research version; SCL, symptom check list; SPSS, statistical package for social sciences; TAU, treatment as usual; WCQ, ways of coping questionnaire
